# Establishment of a novel indicator of pyroptosis regulated gene transcription level and its application in pan-cancer

**DOI:** 10.1038/s41598-023-44700-8

**Published:** 2023-10-20

**Authors:** Jin-Zhou Xu, Qi-Dong Xia, Jian-Xuan Sun, Chen-Qian Liu, Jun-Lin Lu, Meng-Yao Xu, Ye An, Yang Xun, Zheng Liu, Jia Hu, Cong Li, Shao-Gang Wang

**Affiliations:** Department of Urology, Tongji Hospital, Tongji Medical College, Huazhong University of Science and Technology, Wuhan, China

**Keywords:** Cancer, Computational biology and bioinformatics, Genetics, Immunology

## Abstract

Pyroptosis is a type of programmed cell death and plays a dual role in distinct cancers. It is elusive to evaluate the activation level of pyroptosis and to appraise the involvement of pyroptosis in the occurrence and development of diverse tumors. Accordingly, we herein established an indicator to evaluate pyroptosis related gene transcription levels based on the expression level of genes involved in pyroptosis and tried to elaborated on the association between pyroptosis and tumors across diverse tumor types. We found that pyroptosis related gene transcription levels could predict the prognosis of patients, which could act as either a favorable or a dreadful factor in diverse cancers. According to signaling pathway analyses we observed that pyroptosis played a significant role in immune regulation and tumorigenesis and had strong links with other forms of cell death. We also performed analysis on the crosstalk between pyroptosis and immune status and further investigated the predictive potential of pyroptosis level for the efficacy of immunotherapy. Lastly, we manifested that pyroptosis status could serve as a biomarker to the efficacy of chemotherapy across various cancers. In summary, this study established a quantitative indicator to evaluate pyroptosis related gene transcription levels, systematically explored the role of pyroptosis in pan-cancer. These results could provide potential research directions targeting pyroptosis, and highlighted that pyroptosis may be used to develop a novel strategy for the treatment of cancer.

## Introduction

Pyroptosis is a type of programmed cell death mediated by gasdermin family. It features a sequence of morphological changes, including cell swelling, plasma membrane dissolution, and chromatin rupture, and further allows the release of intracellular pro-inflammatory substances, finally triggering the inflammation^1^. Therefore, it is widely entailed in various human diseases, including immune diseases, infectious diseases, and tumors^2–4^.

Increasing attention has been poured into the involvement of pyroptosis in the occurrence and development of diverse tumors. On the one hand, this inflammatory cell death pathway can promote the proliferation and activation of immune cells and facilitate the release of cytokines, which can mediate an antitumor immune function^5^. For example, the overexpression of gasdemin E (GSDME) in cancer cells significantly enhances the number and activity of infiltrating natural killer (NK) cells and CD8 + T lymphocytes within tumors^6^. On the other hand, the chronic inflammatory microenvironment induced by pyroptosis can give rise to inflammatory-tumor transformation and cell carcinogenesis^7^. Specifically, pyroptosis-induced cytokine release, such as IL-1 and IL-18, can promote angiogenesis in tumors, thereby increasing the likelihood of tumorigenesis and metastasis^8^. Besides, high‐mobility group box 1(HMGB1), released by pyroptotic cells, could also promote tumor cell survival via the accumulation of myeloid‐derived suppressor cells^9^. Whether pyroptosis promotes or inhibits tumorigenesis, tumor progression, angiogenesis, and metastasis depend on the clinical characters, tumor types, tumor hallmarks, immune microenvironment, and molecular subtypes^10^.

Moreover, people also showed solicitude for the role of pyroptosis in anti-tumor therapy. Cheng-Cheng et al. found that chemotherapeutic paclitaxel and cisplatin could induce pyroptosis via caspase-3/GSDME activation^11^. Cucurbitacin B was suggested to trigger TLR4/NLRP3/GSDMD-dependent pyroptosis to inhibit tumors^12^. It was also reported that activating pyroptosis could also reverse chemoresistance in lung and pancreatic cancer^13^.

Hence, in this study, we constructed an index to objectively illustrate the status of pyroptosis based on pyroptosis regulator gene expression. We further dissected the relation between pyroptosis and patient survival, clinical features, cancer hallmarks, immune microenvironment, drug sensitivity in different cancer types. This study can provide a computed indicator to evaluate the pyroptosis related gene transcription levels and elaborate the association between pyroptosis and tumors across diverse tumor types. These results may highlight that pyroptosis may be used to develop a novel strategy for the treatment of cancer.

## Results

### Establishment of PPI and the clinical relevance of pyroptosis

**Figure 1:**
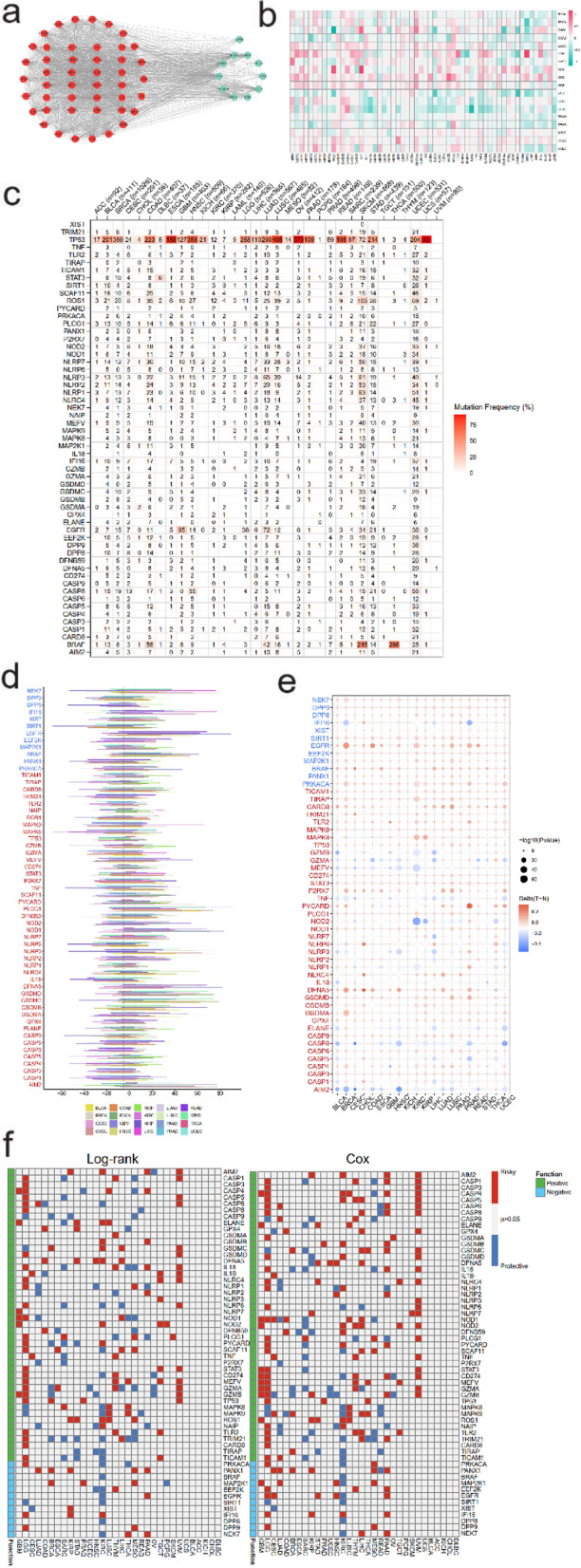
Establishment of PPI and the clinical relevance of pyroptosis. (**a**) Protein–protein interaction network of positive regulator genes (left part with red circles) and negative regulator genes (right part with green circles). (**b**) Ratio of expression of PRGs in tumor tissue to normal tissue after log 2 transformation. (**c**) Heatmap showing the mutation frequencies of PRGs in different cancer types. (**d**) Histogram showing the frequencies of somatic copy number alterations for PRGs in different cancer types. (**e**) Heatmap showing the differential methylation of PRGs in different cancer types. (**f**) Survival relevance of expression levels of PRGs utilizing log-rank test (left part) and cox regression (right part).

After a comprehensive review, 57 genes were determined as pyroptosis regulator genes (PRGs). Positive regulators included AIM2, CASP1, CASP3, CASP4, CASP5, CASP6, CASP8, CASP9, ELANE, GPX4, GSDMA, GSDMB, GSDMC, GSDMD, GSDME, IL18, IL1B, NLRC4, NLRP1, NLRP2, NLRP3, NLRP6, NLRP7, NOD1, NOD2, PJVK, PLCG1, PYCARD, SCAF11, TNF, P2RX7, STAT3, CD274, MEFV, GZMA, GZMB, TP53, MAPK8, MAPK9, ROS1, NAIP, TLR2, TRIM21, CARD8, TIRAP, and TICAM1, while negative regulators included PRKACA, PANX1, BRAF, MAP2K1, EEF2K, EGFR, SIRT1, XIST, IFI16, DPP8, DPP9, and NEK7. The protein–protein interaction network of these genes was illustrated as Fig. 1a and the detailed sample size of each cancer type was showed in Table 1. We compared the average expression levels of these genes in tumor and normal tissues in varied cancer types (Fig. 1b). Further, we illustrate the molecular alterations of each PRG in 20 cancer types, including mutation frequencies (Fig. 1c), frequencies of somatic copy number alterations (Fig. 1d), and differential methylation (Fig. 1e). We also evaluated the survival relevance of these PRGs utilizing log-rank test and cox regression (Fig. 1f). It was observed that the expression levels and survival relevance of positive PRGs and negative PRGs presented heterogeneity in different cancers.

**Table 1:** Included samples from different cancer types in this study.

Cancer types	Abbreviation	Total sample	Tumor sample	Normal sample
Adrenocortical carcinoma	ACC	77	77	0
Bladder urothelial carcinoma	BLCA	426	407	19
Breast invasive carcinoma	BRCA	1212	1099	13
Cervical squamous cell carcinoma and endocervical adenocarcinoma	CESC	309	306	3
Cholangiocarcinoma	CHOL	45	36	9
Colon adenocarcinoma	COAD	331	290	41
Lymphoid neoplasm diffuse large B-cell lymphoma	DLBC	47	47	0
Esophageal carcinoma	ESCA	195	182	13
Glioblastoma multiforme	GBM	171	166	5
Head and neck squamous cell carcinoma	HNSC	564	520	44
Kidney chromophobe	KICH	91	66	25
Kidney renal clear cell carcinoma	KIRC	603	531	72
Kidney renal papillary cell carcinoma	KIRP	321	289	32
Acute myeloid leukemia	LAML	173	173	0
Brain lower grade glioma	LGG	523	523	0
Liver hepatocellular carcinoma	LIHC	421	371	50
Lung adenocarcinoma	LUAD	574	515	59
Lung squamous cell carcinoma	LUSC	548	498	50
Mesothelioma	MESO	87	87	0
Ovarian serous cystadenocarcinoma	OV	427	427	0
Pancreatic adenocarcinoma	PAAD	183	179	4
Pheochromocytoma and paraganglioma	PCPG	185	182	3
Prostate adenocarcinoma	PRAD	548	496	52
Rectum adenocarcinoma	READ	103	93	10
Sarcoma	SARC	264	262	2
Skin cutaneous melanoma	SKCM	470	469	1
Stomach adenocarcinoma	STAD	450	414	36
Testicular Germ cell tumors	TGCT	154	154	0
Thyroid carcinoma	THCA	571	512	59
Thymoma	THYM	121	119	2
Uterine corpus endometrial carcinoma	UCEC	204	181	23
Uterine carcinosarcoma	UCS	57	57	0
Uveal melanoma	UVM	79	79	0
Total		10,534	9807	627

**Figure 2:**
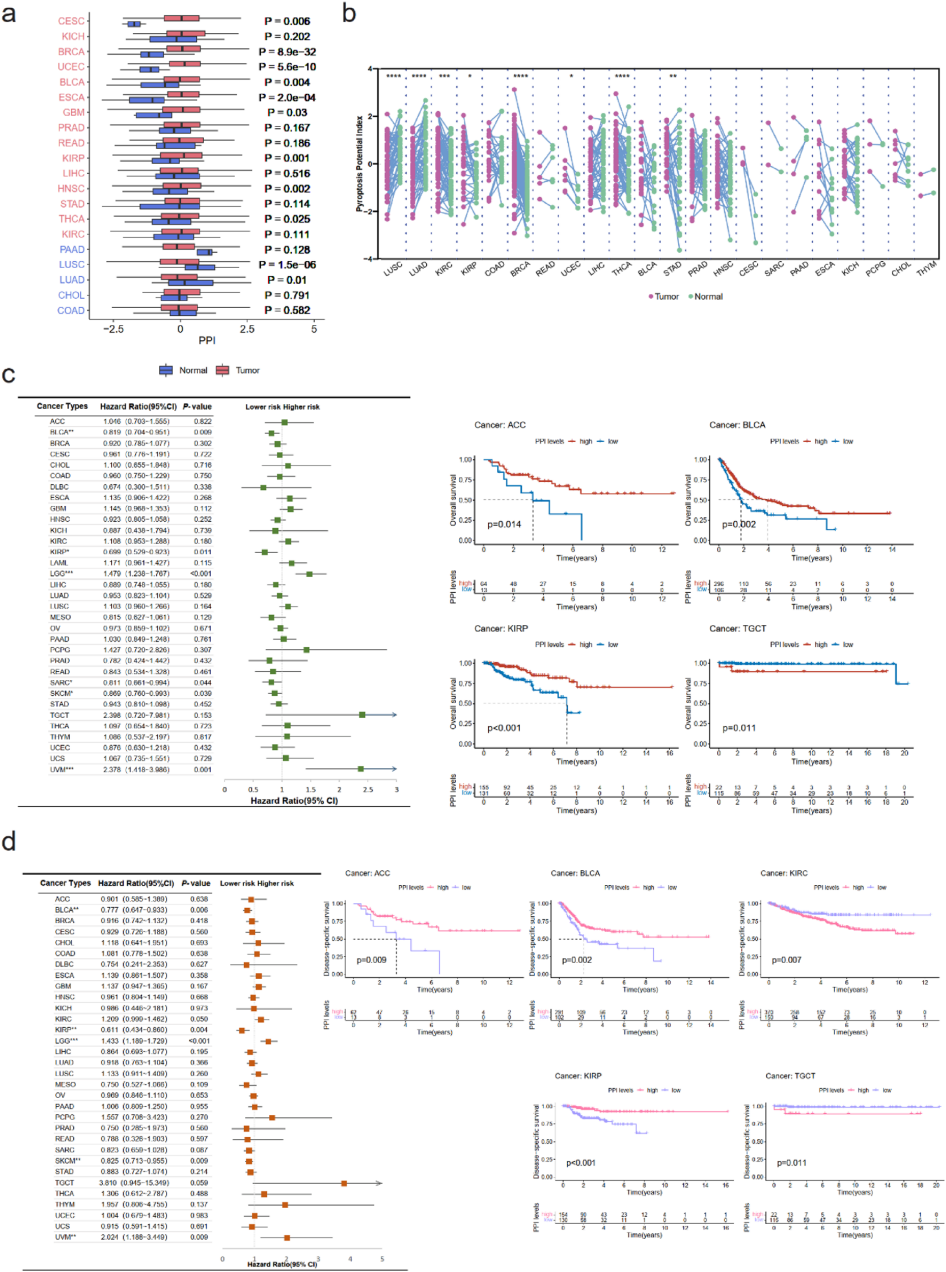
Establishment of PPI and the clinical relevance of pyroptosis. (**a**) Comparation regarding the differences of PPI between tumor (red bars) and normal (purple bars) tissues. (**b**) Differences of PPI between paired tumor (purple) and normal (green) tissues. (**c**) Association between PPI and overall survival measured by hazard ratio. Illustrations of predictive values for overall survival of PPI modeled by Kaplan–Meier survival curve. *p < 0.05, **p < 0.01, ***p < 0.001. (**d**) Association between PPI and disease-specific survival measured by hazard ratio. Illustrations of predictive values for disease-specific survival of PPI modeled by Kaplan–Meier survival curve. *p < 0.05, **p < 0.01, ***p < 0.001.

We then established pyroptosis potential index (PPI) based on the enrichment score of positive regulator genes and negative regulator genes mentioned above. We compared the average PPI between tumor and normal tissues in TCGA. In CESC, BRCA, UCEC, BLCA, ESCA, GBM, KIRP, HNSC, and THCA PPI was significantly higher in tumor tissues, while in LUSC and LUAD PPI was higher in normal tissues (Fig. 2a). We further applied analyses on the differences of PPI in paired tumor and normal tissues (Fig. 2b). We found that in LUSC, LUAD pyroptosis was more activated in normal tissues, and in KIRC, KIRP, BRCA, UCEC, THCA STAD pyroptosis level was higher in tumor tissues.

Then we evaluated the clinical relevance of pyroptosis based on PPI. Concerning overall survival, pyroptosis related gene transcription levels were associated with favorable survival in BLCA, KIRP, SARC, and SKCM and associated with a dreadful survival in LGG and UVM (Fig. 2c). We further performed Kaplan–Meier survival curve analysis between the high- and the low-PPI groups coming out that overall survival of patient was significantly related to pyroptosis activities in most cancer types among 33 cancer types. In patient suffering from ACC, BLCA, KIRP, BRCA, CESC, HNSC, MESO, SARC, SKCM, STAD, a higher pyroptosis degree seemed to promise a beneficial survival. Nevertheless, a higher PPI was predictive of a poor prognosis in TGCT, COAD, GBM, LAML, LUSC, LGG, PCPG, UCS, and UVM (Fig. 2c and Fig. S1a). Similarly, we dissected the relationship between PPI and disease-specific survival (Fig. 2d and Fig. S1b), disease-free survival (Fig. S2a), and progression-free survival (Fig. S2b). Taking all the four survival indexes under consideration, in ACC, BLCA, BRCA, CESC, and KIRP, pyroptosis activation was observed as a favorable survival factor, while in LUSC it might mean a signal of poor prognosis.

To investigate the possible explanations for the correlation between pyroptosis and patient survival, we established the relationship between PPI and clinical characteristics in diverse cancer types, including age, sex, race, histologic grade, and stage (Fig. S3). It was unearthed that in most cancer types, males carried higher pyroptosis related gene transcription levels than females (Fig. S3b). In LGG and KIRC a higher PPI indicated a higher level of grade, which might demonstrate the association to an unfavorable prognosis. However, in UCEC a higher pyroptosis degree meant a greater probability for grade 1 (Fig. S3d). Patient with higher pyroptosis related gene transcription levels was more likely to be diagnosed to be in stage I in ACC, and less likely to be in stage IV in KIRP, which corresponded to the protective role PPI played in survival results (Fig. S3e). These results presented a tool to evaluate the pyroptosis activity and demonstrated a landscape of clinical relevance of pyroptosis in various cancer types.

### Association between pyroptosis and signaling pathways and biological behavior in cancers

**Figure 3:**
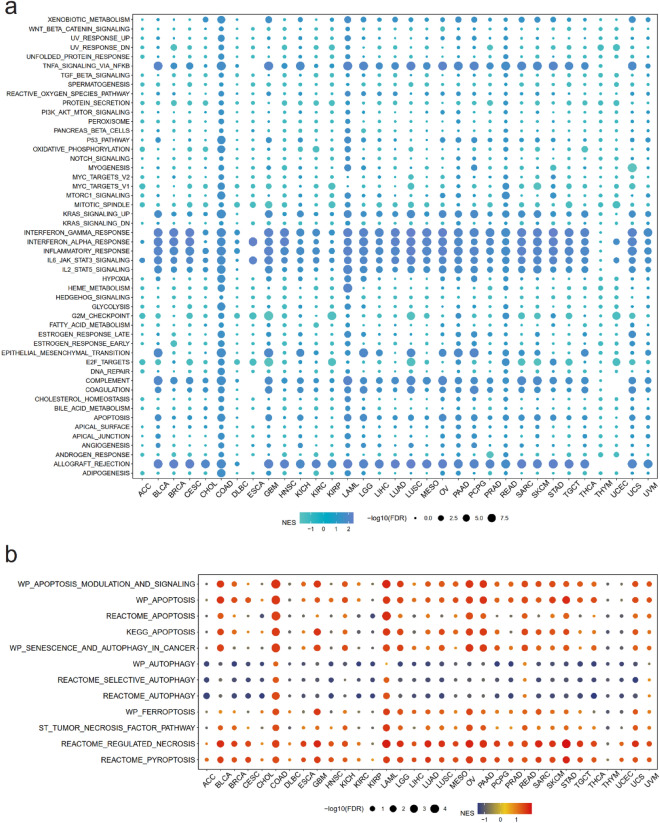
Association between pyroptosis and signaling pathways and biological behavior in cancers. (**a**) Relation between pyroptosis and cancer hallmark pathways in various cancers. (**b**) Relation between pyroptosis and levels of different cell death in various cancers. The color presented normalized enrichment score (NES), and the size of the circle showed false discovery rate (FDR) after − log10 transformation.

To explore the association between pyroptosis and signaling pathways, we applied gene set enrichment analysis (GSEA) of pyroptosis in pan-cancer and first focused on the role pyroptosis played in tumor characteristic pathways. It was noticed that a relatively higher PPI manifested higher normalized enrichment score (NES) in several pathways related to immune reaction, indicating a close link between pyroptosis and these pathways, including TNF-α/NF-κB pathway, INF-α and INF-γ response, inflammatory response, IL-6/JAK/STAT signaling pathway, and complement pathway (Fig. 3a). In addition, according to the mean value of PPI, we divided the samples into high- and low-PPI groups. Based on the results from Kyoto Encyclopedia of Genes and Genomes (KEGG) analyses^14^, we inferred that pyroptosis was related to a few signaling pathways involved in immunomodulation across diverse cancer types, including chemokine signaling pathway, cytokine-cytokine receptor interaction, allograft rejection, and primary immunodeficiency (Fig. S4,5). Gene Ontology (GO) analyses suggested that the PRGs might have the potential to regulate pathways related to cell proliferation, tumorigenesis, and immune response, including chromatin assembly or disassembly, cell cycle transition, immune response regulating cell surface receptor signaling, endothelial cell proliferation, innate immune response, and T cell proliferation (Figs. S6–8).

Furthermore, we established the association between pyroptosis and tumor biological behavior including cell death, tumor metabolism, aging and post translation modification. Through different algorithms, we could conclude that in most cancer types, pyroptosis was positively correlated to apoptosis, ferroptosis and necrosis, and negatively related to autophagy process (Fig. 3b). Concerning metabolism, pyroptosis showed a positive relevance to selenoamino acid metabolism, polyamines metabolism and arachidonic acid metabolism. The association between pyroptosis and metabolism across diverse cancers presented apparent heterogeneity (Fig. S9a). Additionally, we discovered that in several cancer types, pyroptosis presented positive relation with cell aging. Regarding post translation modification, it was revealed that pyroptosis related gene transcription levels were positively associated with ubiquitination and sumoylation. Nevertheless, in COAD and PRAD, PPI presented negative correlation to levels of aging, ubiquitination and sumoylation (Fig. S9b).

### Association between pyroptosis and immune status

**Figure 4:**
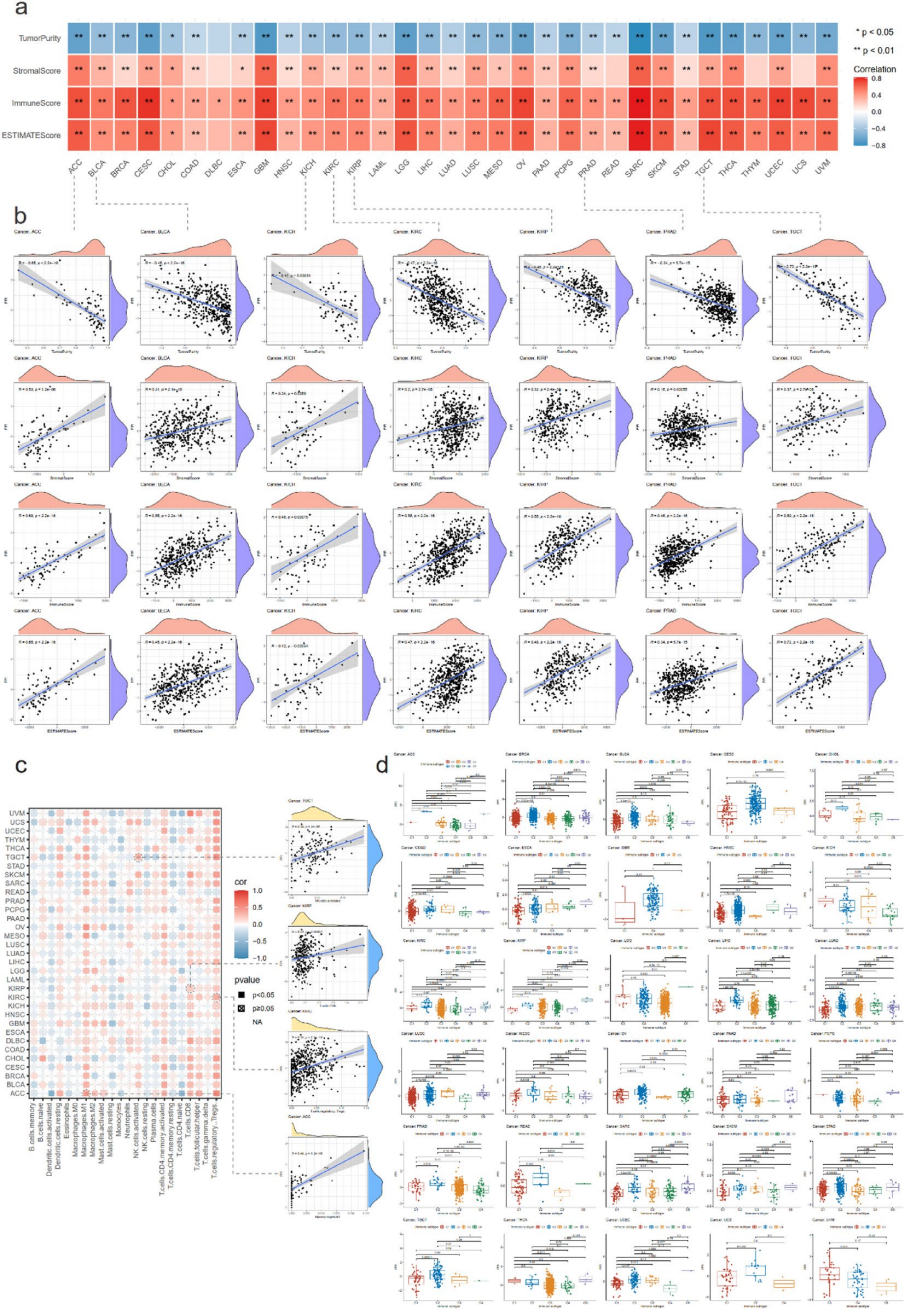
Association between pyroptosis and immune status. (**a**) Evaluation of the correlation of PPI to the tumor immune microenvironment calculated with ESTIMATE package. Colours indicated the correlation coefficients and * demonstrated the significance of the correlation. (**b**) Elaborated correlation between neoplasia of the genitourinary tract and PPI. The irregular shape in the upper (orange) and the right (purple) of each figure indicated the distribution of scores and PPI, respectively. (**c**) Association between PPI and immunocyte infiltration applying CIBERSORT package. Colours indicated the correlation coefficients and shapes demonstrated the significance of the correlation. The right part manifested the correlation of PPI with certain immunocyte in partial genitourinary tract cancers. (**d**) Boxplots illustrating the values of PPI in 6 immune subtypes in pan-cancer.

Results from former analyses indicated that PRGs played essential part in immunoregulation and pyroptosis was also reported to be accompanied by immune reaction, especially alterations in tumor immune microenvironment^15^. Hence, we applied several methods to assess the association between pyroptosis and tumor immune microenvironment status. First, we utilized ESTIMATE package to calculate the tumor purity, stromal score, immune score, and ESTIMATE score in diverse tumors and established the correlation between the scores and PPI. In most cancer types, PPI was significantly positively related to stromal score, immune score, and ESTIMATE score, and was negatively related to tumor purity. The correlation between PPI and immune microenvironment scores was also correspond with the trend in DLBC, READ, THYM, and UCS, although not significant in certain indexes (Fig. 4a). Concerning tumor types of the genitourinary tract (ACC, BLCA, KICH, KIRC, KIRP, PRAD, TGCT), PPI could also promisingly represent the cancer microenvironment conditions. The correlation coefficients between PPI and tumor purity were − 0.65, − 0.45, − 0.42, − 0.47, − 0.48, − 0.34, − 0.72 in ACC, BLCA, KICH, KIRC, KIRP, PRAD, TGCT, respectively. Meanwhile, the coefficients between PPI and immune score were, respectively, 0.69, 0.55, 0.46, 0.56, 0.55, 0.46, 0.69 in ACC, BLCA, KICH, KIRC, KIRP, PRAD, TGCT. All *p* values < 0.001 (Fig. 4b). Detailed correlations between PPI and tumor purity, stromal score, immune score, and ESTIMATE score in other tumor types were indicated in Fig. S10.

We next applied CIBERSORT algorithm to explore the association between pyroptosis and immune microenvironment and to investigate the potential of PPI to be representative of immune microenvironment status. It was observed that PPI could to a large degree illustrate the tumor microenvironment from the perspective of the evaluation of the immunocyte infiltration. Pyroptosis related gene transcription levels showed a positive correlation with M1 macrophages, CD8 + T cells, and regulatory T cells (Tregs) in various cancer types. Furthermore, regarding tumor types of the genitourinary tract, PPI was significantly positive associated with M1 macrophages infiltration in ACC (R = 0.44), Tregs infiltration in KIRC (R = 0.36), CD8 + T cells infiltration in KIRP (R = 0.21), and activated NK cells infiltration in TGCT (R = 0.34), respectively (Fig. 4c). All *p* values < 0.001. Elaborated analyses on the relationship between PPI and immunocyte infiltration in diverse tumors were presented in Fig. S11.

To characterize the association between intratumoral immune states and pyroptosis, we then developed boxplots illustrating PPI in 6 immune subtypes in diverse cancers (Fig. 4d)^16^. In pan-cancer, it presented distinct tendency to be under different circumstances of immune subtypes. Concerning tumor types of the genitourinary tract, C3 (Inflammatory) subtypes took the dominance in KICH, KIRC, KIRP, and PRAD. In BLCA and TGCT, C1 (Wound Healing) and C2 (IFN-γ Dominant) subtypes were in the majority. It was also observed that various types of tumors displayed significant variation within the association between PPI and immune subtypes. In quite a few cancer types, PPI in C2 subtype held the advantage, which had the highest M1/M2 macrophage polarization, a strong CD8 signal, and the greatest TCR diversity. In some cancer types C2 subtype ranked not the first, which might be attributed to the small amounts of cases. In addition, in LGG C3, C4 (Lymphocyte Depleted) subtypes had higher PPI. These outcomes established the association between pyroptosis and immune status and also suggested the potential predictive values of PPI to evaluated the immune microenvironment conditions in various cancers.

### Relation between pyroptosis and molecular features in cancers

**Figure 5:**
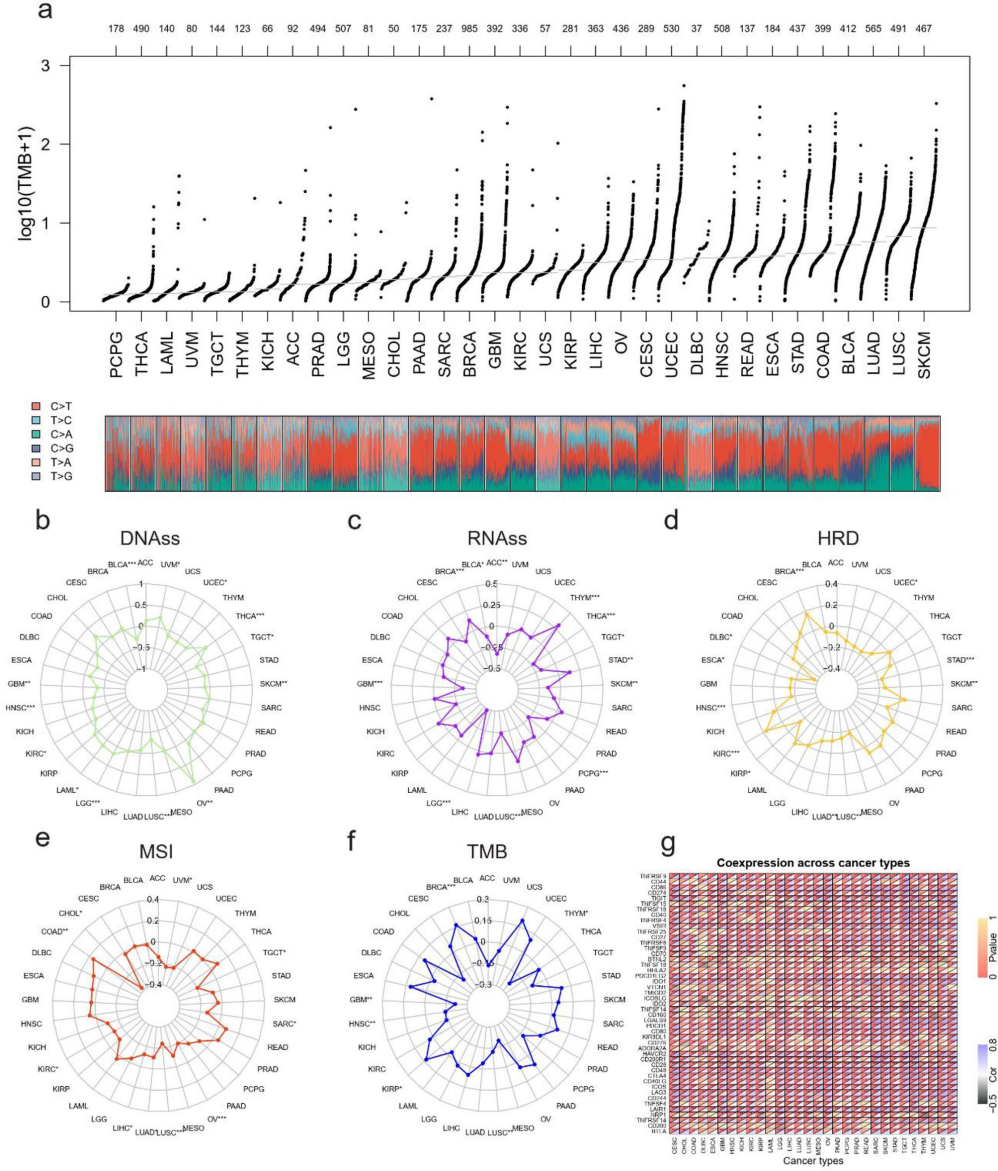
Relation between pyroptosis and molecular features in cancers. (**a**) Landscape of TMB in pan-cancer. Results were presented after log10 transformation. Numeric characters in the upper figure indicated the numbers of the cases. Relation between PPI and tumor characteristics in various tumors types were established in radar maps (**b**–**f**). Tumor stemness were measured based on mRNA expression (RNAss, **b**) and DNA methylation (DNAss, **c**). Genome instability were evaluated by homologous recombination deficiency (HRD, **d**), microsatellite instability (MSI, **e**), tumor mutation burden (TMB, **f**). (**g**) Correlation between PPI and mRNA expression of acknowledged immune checkpoints in different tumor. The upper triangle of each tile demonstrates the *p* value, the lower triangle indicates coefficient applying Pearson correlation test. *p < 0.05, **p < 0.01, ***p < 0.001.

To elaborate correlation between pyroptosis and cancer molecular features and to determine the potential of PPI to predict the immunotherapeutic response, we underwent the following analyses. We conducted research on tumor stemness based on mRNA expression (RNAss) and DNA methylation (DNAss). Taking both tumor stemness scores into consideration, a higher PPI was significantly associated with lower cancer stem cell‐like properties in BLCA, TGCT, SKCM, LUSC, and GBM (Fig. 5b,c).

We further drew the association between pyroptosis activation and genome instability. PPI was positively related to homologous recombination deficiency (HRD) in BRCA and KIRC and negatively related to HRD in ESCA, HNSC, KIRP, LUAD, LUSC SKCM, STAD, and UCEC (Fig. 5d). Furthermore, pyroptosis degree presented a negative relation to the levels of microsatellite instability (MSI) in 9 cancer types including CHOL, KIRC, LIHC, LUAD, LUSC, OV, SARC, TGCT, UVM. Whereas, COAD was the only cancer type, where pyroptosis activation was connected to a higher level of MSI (Fig. 5e). We also poured consideration on the tumor mutation burden (TMB). Figure 5a illustrated the landscape of TMB in pan-cancer and we found that pyroptosis related gene transcription levels were positively associated with TMB in BRCA and KIRP, and were negatively associated with TMB in GBM, HNSC, LUSC, THYM (Fig. 5f).

Pyroptosis was also manifested to be entailed in therapeutic effect and have a crosstalk with checkpoint inhibitors^17^. Accordingly, we next determined the correlation between PPI and mRNA expression of acknowledged immune checkpoints in multiple tumors. Correlation analyses between PPI and checkpoint gene expression unearthed a high correlation of pyroptosis related gene transcription levels with tumor necrosis factor (TNF)-related immune genes (TNFRSF 4, 8, 9, 14, 18) and T lymphocyte-related immune genes (CD28, CD80, CD86) in various cancer types. The results also indicated that pyroptosis activities showed a close relation with most immune checkpoints. PD1-related checkpoints were highly related to pyroptosis activities in different cancer types, including Programmed Cell Death 1 (PD-1, PDCD1), Programmed Cell Death 1 Ligand 1 (PD-L1, CD274), and Programmed Cell Death 1 Ligand 2 (PDCD1LG2). Additionally, PPI was associated significantly with more than 20 immune checkpoint genes in most cancer types except for in CHOL, DLBC, and LAML. Furthermore, in those types that had the largest TMB including SKCM, LUSC, and LUAD, PPI possessed an overwhelmingly close link to the expression of immune checkpoints (Fig. 5g).

### Treatment relevance of pyroptosis

**Figure 6:**
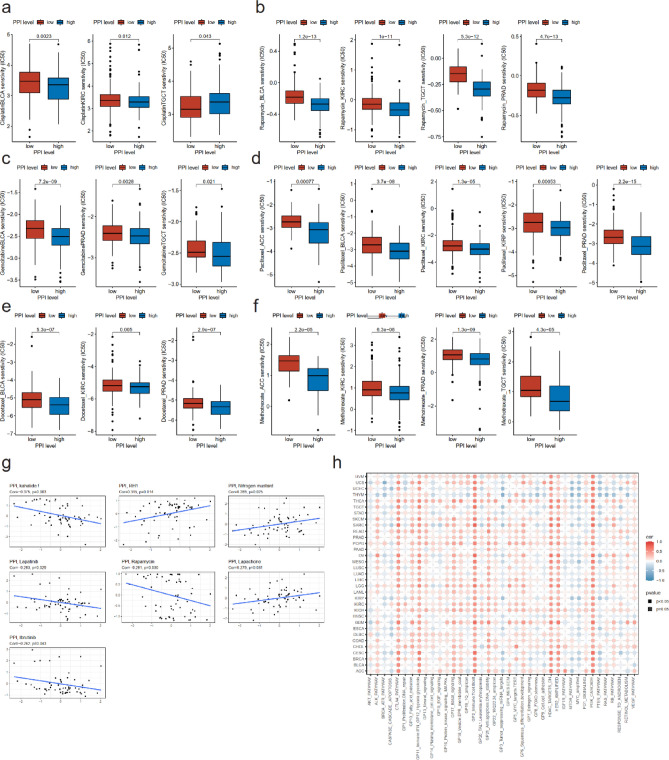
Treatment relevance of pyroptosis. Half maximal inhibitory concentration (IC50) of cisplatin (**a**), rapamycin (**b**), gemcitabine (**c**), paclitaxel (**d**), docetaxel (**e**), and methotrexate (**f**) grouped by low- or high-PPI in partial genitourinary tract cancers. (**g**) Correlation between PPI and drug sensitivity from CellMiner database. (**h**) Association between PPI and treatment pathway scores in pan-cancer. Colours indicated the correlation coefficients and shapes demonstrated the significance of the correlation.

It was indicated that chemotherapy could function by inducing pyroptosis^18^. Accordingly, we applied drug sensitivity analyses to further explore the probable effects of pyroptosis on drug response. We illustrated the drug response by half maximal inhibitory concentration (IC50) of cisplatin, rapamycin, gemcitabine, paclitaxel, docetaxel, sorafenib, and methotrexate grouped by values of PPI. Regarding tumor types of the genitourinary tract (ACC, BLCA, KICH, KIRC, KIRP, PRAD, TGCT), a lower pyroptosis activation in BLCA and KIRC indicated a significant lower sensitivity to cisplatin while could demonstrate a higher sensitivity to cisplatin in TGCT. Additionally, cisplatin is a common therapy for BLCA and TGCT (Fig. 6a). A higher PPI was related to higher effectiveness of rapamycin treatment in BLCA, KIRC, TGCT, and PRAD (Fig. 6b). A higher PPI was significantly associated with lower CI50 of gemcitabine in BLCA, PRAD and TGCT, which was a common treatment of intravesical chemotherapy after operation for BLCA (Fig. 6c). For paclitaxel, in ACC, BLCA, KIRC, KIRP, and PRAD, a higher pyroptosis level could promise a higher chemosensitivity (Fig. 6d). In KIRC and PRAD, a higher pyroptosis activity manifested a more sensitive response to docetaxel and methotrexate (Fig. 6e,f). Overlooking the correlation between PPI and drug response in pan-cancer, higher levels of pyroptosis activation evidenced to be more responsive to diverse chemotherapeutic drugs (Fig. S12).

We then displayed the scatter plots drawing the correlation between drug sensitivity and pyroptosis activities in CellMiner database. Notably, PPI was positively correlated with sensitivity of Ginsenoside (Rh1) (Pearson correlation coefficient = 0.315, *p* = 0.014), nitrogen mustard (Pearson correlation coefficient = 0.289, *p* = 0.025), and lapachone (Pearson correlation coefficient = 0.279, *p* = 0.031), and negatively correlated with sensitivity of kahalide f (Pearson correlation coefficient = − 0.375, *p* = 0.003), lapatinib (Pearson correlation coefficient = − 0.283, *p* = 0.029), rapamycin (Pearson correlation coefficient = − 0.281, *p* = 0.030), and ibrutinib (Pearson correlation coefficient = − 0.262, *p* = 0.043) (Fig. 6g).

It has been widely discussed that pyroptosis could be induced via several signaling pathways including PI3K/AKT signaling pathway^19^, and HDAC8-mediated reprogramming^20^. Hence, we further developed the association between PPI and treatment pathway scores in pan-cancer. Pyroptosis degree was in most cancer types significantly positively related to cytotoxic T-lymphocyte antigen 4 (CTLA4) pathway, interferon (IFN) pathway, hypoxia and glycolosis pathway, T cell and B cell pathway, histone deacetylase (HDAC) pathway, human epidermal growth factor receptor 2 (HER2) pathway, and phosphatidylinositol 3 kinase (PI3K) pathway. These results could provide potential therapeutic targets and tailored treatment strategies in diverse cancer types (Fig. 6h).

## Discussion

Pyroptosis plays a dual pro-tumor or anti-tumor role. Brian et al. revealed that pyroptosis could serve as a suppressor in colitis-associated colorectal cancer^21^. Qing et al.^22^ reported that 17β-estradiol-induced activation of the NLRP3 inflammasome leaded to pyroptosis, which proved to be protective against hepatocellular carcinoma. Nevertheless, it was also indicated that pyroptotic activity was greater in tumor cells, and that pyroptosis-induced chronic inflammatory microenvironment could contribute to the immune escape and local immunosuppression^23^. The effect of pyroptosis on tumorigenesis and tumor progression in various tumor types presented to be complex and remained blurry. Therefore, we generated this systematic study to draw the association between pyroptosis and tumor across various cancer types.

In this study, we first distilled 57 PRGs from 2 reviews^24,25^. We applied analyses on the expression and survival relevance of these PRGs in diverse cancers. According to the reported role of these genes in pyroptosis, we further employed ssGSEA to establish an objective and quantitative index PPI to characterize pyroptosis related gene transcription levels, and conducted analyses based on PPI. PPI was higher in tumor than in normal tissue in some cancer types and showed an opposite trend in other cancer types. We found in lung cancers including LUAD and LUSC, PPI was higher in normal tissue than in tumor. Consistently, it was reported that p53 could suppress tumor growth by prompting pyroptosis in non-small-cell lung cancer and activating miR-335/SOD2/ROS signal pathway mediated pyroptotic cell death could also inhibit development of non-small cell lung cancer^26,27^. Concerning patient survival, histologic grade and stage, the outcomes of clinical relevance analyses of pyroptosis corresponded to its role as a double-edged sword in pan-cancer: pyroptosis could act as either a favorable or a dreadful factor in diverse cancers^28^. Notably, males tended to have higher pyroptosis related gene transcription levels in females, and distinct races also had different tendency for pyroptosis related gene transcription levels across diverse cancer types. These outcomes were suggestive of the indispensable to tailor the treatment strategies to the patient characteristics and cancer types when applying pyroptosis to oncotherapy.

To uncover the signaling pathway enrichment of PRGs, we used GSEA to discover that pyroptosis was highly related to pathways entailed in immunomodulation and tumorigenesis, and predictably associated with inflammation related pathways which coincided with former publications^29^. Furthermore, we unearthed how pyroptosis got involved in tumor biological behavior. An emerging form of inflammatory cell death, PANoptosis, has been recently paid more attention to, and indicated a strong association among different modes of cell death^30^. Accordingly, we first established the association between pyroptosis and other forms of cell death and the results demonstrated positive correlations among pyroptosis, apoptosis, ferroptosis, and regulated necrosis. Interestingly, pyroptosis presented a negative correlation with autophagy in most cancer types. Ming-Yong et al. reported that adrenomedullin could protected Leydig cells from pyroptosis by activating autophagy, which corresponded to our results^31^. Nevertheless, it was also indicated that inhibiting the autophagic-inflammasomal pathway could attenuate pyroptosis in liver disease^32^. The contradiction might be explained by the diverse pathways of pyroptosis and the heterogeneity in various diseases, and further researches could give insight into the complex interaction between pyroptosis and autophagy. Recent publications revealed that pyroptosis could promote inflamm-aging^33^. Hence, we performed the analysis on the association between PPI and aging-related gene set, resulting in a negative correlation between pyroptosis and aging. We further established the association between pyroptosis and post transcription modification to discover that higher PPI could indicate suppression of ubiquitination and sumoylation. Ubiquitination and sumoylation of pyroptosis-related proteins could inhibit pyroptosis^34,35^. However, a comprehensive understanding of the interaction of ubiquitination and sumoylation with pyroptosis concerning the whole cellular condition relied on further researches. These results could illustrate a landscape of the pyroptosis in signaling pathways and cell biological behavior in diverse cancer types.

The outcomes from signaling pathway analyses indicated that pyroptosis played a part in immune regulation, and pyroptosis was considered to be accompanied with alterations of tumor immune microenvironment^6^. Synthetic polymer induced pyroptosis was reported to promote immunogenic response and initiate robust antineoplastic immunity^36^. Accordingly, we next established the association of pyroptosis related gene transcription levels with immune status in varied cancers. Utilizing ESTIMATE package, we discovered that pyroptosis related gene transcription levels were significantly positively correlated to ESTIMATE score and negatively related to tumor purity. Additionally, through CIBERSORT package, it was uncovered that pyroptosis showed a positive correlation with CD8 + T cells and M1 macrophages across various cancer types. The interaction between pyroptosis and immunocyte infiltration has been investigated in several publications and could support our discoveries. It was manifested that GSDMD was crucial for an optimal CD8 + T cells response to cancer cells and the upregulation of gasdermin protein could also encourage the infiltration of M1 macrophages and CD8 + T cells^5,37^. Furthermore, after immune subtype analyses we found that in most cancer types, C2 subtype, which had the highest M1/M2 macrophage polarization and a strong CD8 signal, carried the highest pyroptosis level, which corresponded to former results. These results could add to the burgeoning understanding about the crosstalk between pyroptosis and tumor immune reaction, and provide a guideline for further mechanism researches on the reciprocal action of pyroptosis with tumor microenvironment and immunocyte infiltration.

Since pyroptosis was tightly linked to immune status, we then attempted to unearth whether pyroptosis related gene transcription levels could guide immunotherapy. Programmed Cell Death 1 (PD-1) and Programmed Cell Death 1 Ligand 1 (PD-L1) were demonstrated to be highly related to pyroptosis in former studies. The inflammatory microenvironment created via pyroptosis could facilitate the sensitivity of tumor cells to PD-1 related treatment. When treated with PD-1 antibody, expression of pyroptosis related-gene including NLRP3, Caspase4, and GSDMD in macrophages were found to significantly decrease^38^. Meanwhile, PD-L1 was suggested to in turn regulate gasdermin C expression and switch apoptosis to pyroptosis in cancer cells and further to facilitate tumor necrosis^39^. Therefore, we analyzed the association between pyroptosis and cancer molecular features. We observed that pyroptosis activation was negatively correlated to TMB and MSI in several tumor types while was positively linked to PD1-related checkpoints expression. A recent large cohort research reported that TMB, MSI and PD-L1 expression yield substantially discrepancies^40^. Consequently, it was essential that these biomarkers should be combined when appraising the relation with pyroptosis and when assessing the patient’s potential benefit from immune therapy across diverse cancer types.

Lastly, we assessed clinical application prospect of pyroptosis especially concentrating on the medicine treatment relevance of pyroptosis in pan-cancer. We discovered that higher pyroptosis related gene transcription levels could guarantee higher sensitivity to common chemotherapeutics in various cancers. Chemotherapy drugs was reported to trigger pyroptosis to generate antitumor effect and a higher PPI could demonstrated an increased excitability of pyroptosis^41^. In addition, we determined the association between PPI and common treatment pathways coming out that pyroptosis related gene transcription levels was tightly linked to several signaling pathways including CTLA4, PI3K, HER2, HDAC and IFN pathways. These outcomes on the one hand could facilitate further researches on the therapeutic targets related to pyroptosis, since overcoming the escaping mechanism of apoptosis in tumor cells by triggering other cell death programs could promisingly promote cancer therapeutics^42^. On the other hand, appraisal of pyroptosis status could serve as a biomarker to screen potential beneficiaries from cancer treatment and could contribute to guide clinical strategies.

This study still harbors several limitations. First, PRGs we extracted from current reviews may not be able to cover all genes involved in pyroptosis activity. More studies of genetic levels on pyroptosis are warranted. Furthermore, we established PPI depending on transcription level quantifications regardless of regulations of translation or modification levels, which was much demanding to comprehensively reflect the biological function. Moreover, besides pyroptosis, these genes can also contribute to other biological processes, which adds to the inaccuracies of PPI to evaluate the pyroptosis activities. Meanwhile, due to the limited information, it was almost impossible to take cancer subtypes into consideration. In addition, this study was observational and retrospective. It was arduous to eliminate the inevitable bias and further prospective researches can preferably update and polish up our discoveries.

## Conclusion

This study developed a computational metric for assessing pyroptosis related gene transcription levels and elucidating the correlation between pyroptosis and tumors. These results could facilitate further explorations into molecular mechanisms and provide insights into potential therapeutic targets for the future.

## Materials and methods

### Data sources

A total of 10,355 samples’ transcriptional data in fragments per kilobase of exon model per Million mapped fragments were downloaded from Xena_TCGA_PAN-CANCER_TOIL RSEM fpkm datasets, their corresponding detail clinical information and immune subtypes were obtained from UCSC_Xena. Molecular signature and pathway activity including tumor stemness indices^43^ (mRNA expression [RNAss], n = 10,876 and DNA methylation [DNAss], n = 9603), homologous recombination deficiency^44^ (HRD) score (n = 10,647), and gene programs (n = 10,844) were also retrieved from UCSC_Xena_TCGA_PAN-Cancer. Tumor mutation burden^45^ (TMB, n = 10,114) were calculated according to the single nucleotide variation profiles of pan-cancer. Microsatellite instability (MSI, n = 10,415) were obtained from the supplementary files of the previous study^46^. Besides, NCI-60 cell lines contain 60 human cancer cell lines from 9 different tissue types^47^. Both transcriptional profiles and the drug sensitivity of all cell lines were downloaded from the CellMiner database. Cell deaths related gene sets, cell senescence-related gene sets, cell metabolism-related gene sets, and Hallmark gene sets of cancer were all retrieved and downloaded from the molecular signature database.

Based on two previous reviews, we collected all the genes reported to be explicitly associated with pyroptosis as pyroptosis regulator genes (PRGs)^24,25^. They were a total of 57 PRGs including Absent In Melanoma 2 (AIM2), Caspase 1 (CASP1), Caspase 3 (CAS3), Caspase 4 (CAS4), Caspase 5 (CAS5), Caspase 6 (CAS6), Caspase 8 (CAS8), Caspase 9 (CAS9), Elastase, Neutrophil Expressed (ELANE), Glutathione Peroxidase 4 (GPX4), Gasdermin A(GSDMA), Gasdermin B (GSDMB), Gasdermin C (GSDMC), Gasdermin D (GSDMD), Gasdermin E (GSDME), Interleukin 18 (IL18), Interleukin 1 Beta (IL1B), NOD-like receptor (NLR)Family CARD Domain Containing 4 (NLRC4), NLR Family Pyrin Domain Containing 1 (NLRP1), NLR Family Pyrin Domain Containing 2 (NLRP2), NLR Family Pyrin Domain Containing 3 (NLRP3), NLR Family Pyrin Domain Containing 6 (NLRP6), NLR Family Pyrin Domain Containing 7 (NLRP7), Nucleotide Binding Oligomerization Domain Containing 1 (NOD1), Nucleotide Binding Oligomerization Domain Containing 2 (NOD2), Pejvakin (PJVK), Phospholipase C Gamma 1 (PLCG1), PYD And CARD Domain Containing (PYCARD), SR-Related CTD Associated Factor 11 (SCAF11), Tumor Necrosis Factor (TNF), Purinergic Receptor P2X 7 (P2RX7), Signal Transducer And Activator Of Transcription 3 (STAT3), PD-L1 (CD274), MEFV Innate Immunity Regulator Pyrin (MEFV), Granzyme A (GZMA), Granzyme B (GZMB), Tumor Protein P53 (TP53), Mitogen-Activated Protein Kinase 8 (MAPK8), Mitogen-Activated Protein Kinase 9 (MAPK9), ROS Proto-Oncogene 1 (ROS1), NLR Family Apoptosis Inhibitory Protein (NAIP), Toll Like Receptor 2 (TLR2), Tripartite Motif Containing 21 (TRIM21), Caspase Recruitment Domain Family Member 8 (CARD8), TIR Domain Containing Adaptor Protein (TIRAP), Toll Like Receptor Adaptor Molecule 1 (TICAM1), Protein Kinase CAMP-Activated Catalytic Subunit Alpha (PRKACA), Pannexin 1 (PANX1), B-Raf Proto-Oncogene (BRAF), Mitogen-Activated Protein Kinase Kinase 1 (MAP2K1), Eukaryotic Elongation Factor 2 Kinase (EEF2K), Epidermal Growth Factor Receptor (EGFR), Sirtuin 1 (SIRT1), X Inactive Specific Transcript (XIST), Interferon Gamma Inducible Protein 16 (IFI16), Dipeptidyl Peptidase 8 (DPP8), Dipeptidyl Peptidase 9 (DPP9), NIMA Related Kinase 7 (NEK7).

### Establishment of the pyroptosis potential index (PPI) in pan-cancer

Having obtained the pyroptosis regulator genes, we firstly systematically investigated the differential expression status of these PRGs between normal tissue and tumor tissue in 18 cancer types that have normal tissues. Then the prognostic value of these PRGs were examined by both log-rank tests and univariate cox regression. Following this, PRGs were input into the STRING database to construct the protein–protein interaction network of these PRGs. Then we downloaded the network and visualized it by the Cytoscape version 3.8.2. Subsequently, we wondered if we could establish a comprehensive parameter to evaluate the individual pyroptosis level according to the expression status of these PRGs. Thus, we performed single-sample gene set enrichment analysis (ssGSEA) by R package “GSVA” in these pan-cancer samples^48^. Notably, the positive and negative regulators genes were used to conduct ssGSEA separately, and we can obtain two different enrichment scores (ES), one for the positive and another for the negative regulators. Finally, the pyroptosis potential index (PPI) was defined according to the follow formula:

$$Pyroptosis\,\,Potential \,\,Index\, = \,ES\,\left( {positive regulators} \right)\, - \,ES\left( {negative\,\, regulators} \right)$$

Then each sample obtained a corresponding PPI according to their transcriptional profiles^49^.

### Differential pyroptosis related gene transcription levels, prognostic values, and related pathways of PPI

The PPI between normal tissue and tumor tissue in different cancer types were compared by the Wilcoxon tests. Following this, we further investigated the prognostic values of PPI in different cancer types by both univariate cox regression and log-rank tests. Finally, we wondered about the mechanism between differential pyroptosis related gene transcription levels. Thus, we divided samples in each cancer type into high-/ low-PPI groups by the medium PPI value in each cancer type. Then performed GSEA analysis by R package “clusterProfiler” to investigate the differential enhanced functions or pathways between high-/ low-PPI levels^50^. Besides, to further explore the pyroptosis related functions or pathways, we selected the samples with the top 30% PPI and the bottom 30% PPI in each cancer type, then performed GSEA analysis using Hallmark gene sets, cell deaths related gene sets, cell senescence-related gene sets, and cell metabolism-related gene sets. Saved the normalized enrichment score (NES) and corresponding false discovery rate (FDR), visualized these in the bubble plots.

### PPI and immunity in pan-cancer

We carried out two different methods, including ESTIMATE and CIBERSORT, to systematically describe the immunity status of each sample in pan-cancer. ESTIMATE algorithm was performed to describe the tumor micro-environment by four parameters; Tumor purity, Stromal score, Immune score, and ESTIMATE scores^51^. CIBERSORT algorithm was used to quantify the detailed infiltration status of 22 different types of immune cells^52,53^. Finally, Spearman correlation tests were conducted to investigate the correlation between immunity status and PPI levels in each cancer types. In addition, we also compared the differences in PPI levels between different immune subtypes and other clinicopathological features by Wilcoxon tests to further verify the correlation between immunity and PPI levels.

### PPI and molecular features in pan-cancer

Tumor mutation burden (TMB), defined as nonsynonymous mutations per megabase (Mb), was calculated by the single nucleotide variation profiles of pan-cancer. Then we separately performed Spearman correlation tests between PPI and TMB, MSI, DNAss, RNAss, HRD scores, and expression of 48 immune checkpoints in pan-cancer types.

### PPI and drug sensitivity in pan-cancer and cancer cell lines

Samples were divided into high-/ low-PPI groups. Then, the ProPhetic algorithm was carried out to evaluate the drug sensitivity (IC50) of commonly used chemotherapy or targeted therapy drugs, including cisplatin, rapamycin, gemcitabine, paclitaxel, docetaxel, methotrexate, and sorafenib^54,55^. Then the drug sensitivity was compared between high or low PPI levels in each cancer type. Besides, PPI of all NCI-60 cell lines were also evaluated by the same method based on the CellMiner database (https://discover.nci.nih.gov/cellminer/home.do). Then the correlation between drug sensitivity to the different anti-tumor drugs and PPI levels in these cell lines were examined to further verify the correlation between PPI levels and drug sensitivity. Finally, we explored the correlation between PPI and drug targets score, which may help to comprehensively understand the potential mechanism behind the correlation between drug sensitivity and PPI and may contribute to developing potential novel drugs targeting both pyroptosis pathway and traditional therapies drug targets.

### Statistics

Log-rank test and cox regression were used in the survival analysis. Wilcoxon tests were used to verify the correlation between immunity and PPI levels. Chi-squared tests and Student's t-tests were applied for variance analyses. Correlation analysis was performed using Spearman correlation analysis. ProPhetic algorithm was carried out to evaluate the drug sensitivity. CIBERSORT algorithm was used to explore the immune microenvironment. R software (version 4.0.3) was used to perform all the statistical analyses. All *p* values were two-tailed. A *p* value < 0.05 was considered statistically significant.

### Supplementary Information

Supplementary Figures.

Supplementary Tables.

## Data Availability

The datasets supporting the conclusions of this article are available in the Xena_TCGA_PAN-CANCER_TOIL RSEM fpkm datasets, (https://xenabrowser.net/datapages/?dataset=tcga_RSEM_gene_fpkm&host=https%3A%2F%2Ftoil.xenahubs.net&removeHub=https%3A%2F%2Fxena.treehouse.gi.ucsc.edu%3A443); UCSC_Xena_TCGA_PAN-Cancer (https://xenabrowser.net/datapages/?cohort=TCGA%20Pan-Cancer%20(PANCAN)&removeHub=https%3A%2F%2Fxena.treehouse.gi.ucsc.edu%3A443), CellMiner database (https://discover.nci.nih.gov/cellminer/home.do), molecular signature database (https://www.gsea-msigdb.org/gsea/msigdb/index.jsp). The dataset supporting the conclusions of this article is included within the article and its additional file.

## Abbreviations

RNAss: MRNA expression
DNAss: DNA methylation
HRD: Homologous recombination deficiency
TMB: Tumor mutation burden
MSI: Microsatellite instability
PRGs: Pyroptosis regulator genes
PPI: Pyroptosis potential index
ssGSEA: Single-sample gene set enrichment analysis
ES: Enrichment scores
NES: Normalized enrichment score
FDR: False discovery rate
Tregs: Regulatory T cells

## Code availability

Original code can be obtained from the corresponding author upon reasonable request.
