# Supplementary material for: Establishment of a novel indicator of pyroptosis regulated gene transcription level and its application in pan-cancer
Source: Sci Rep. 2023 Oct 20;13:17911. doi: 10.1038/s41598-023-44700-8 (PMC10589244; doi:10.1038/s41598-023-44700-8)
Supplement: Supplementary file 1 — Supplementary Figures. [file 41598_2023_44700_MOESM1_ESM.docx]

sFigure


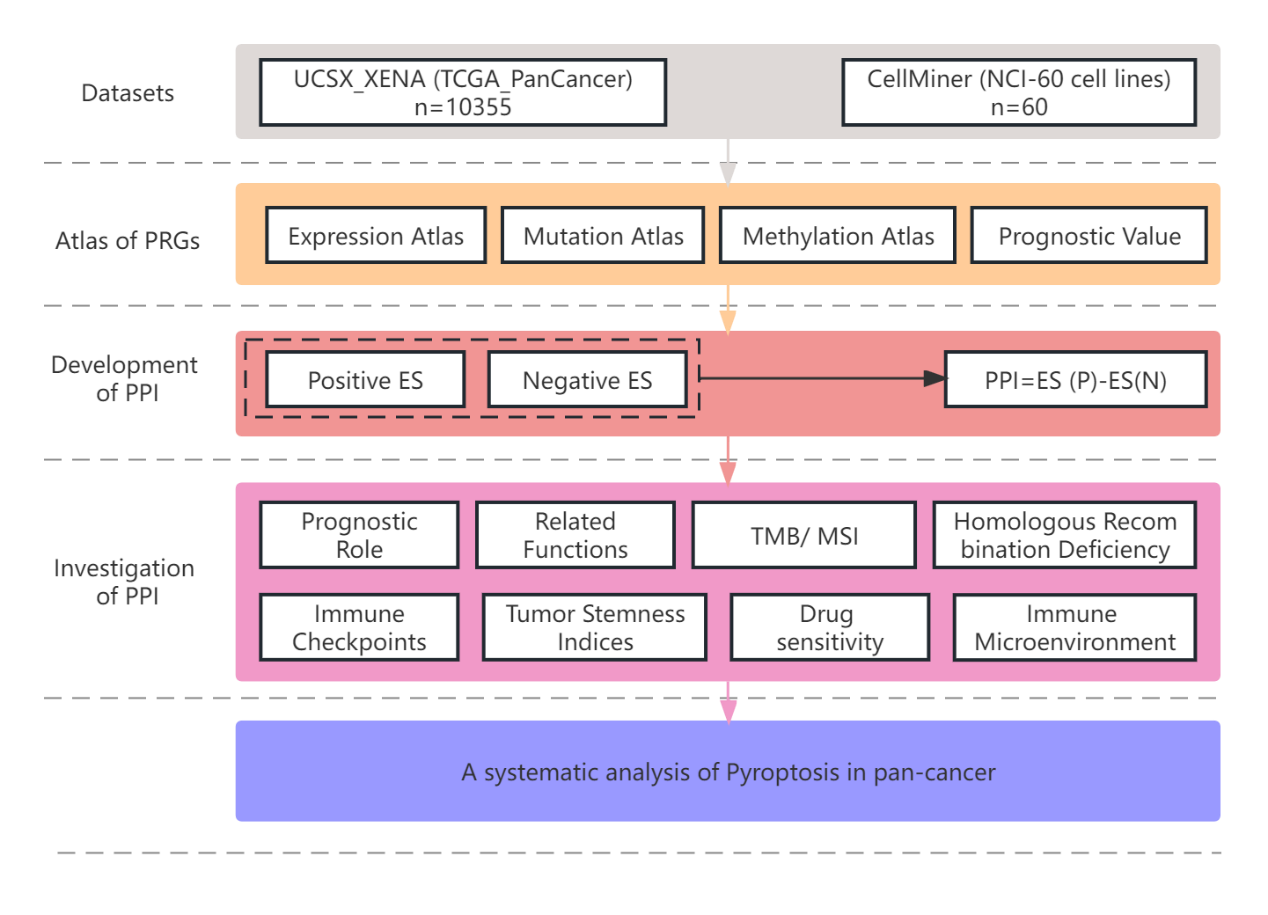


sFigure 1

The flow chart of the study.


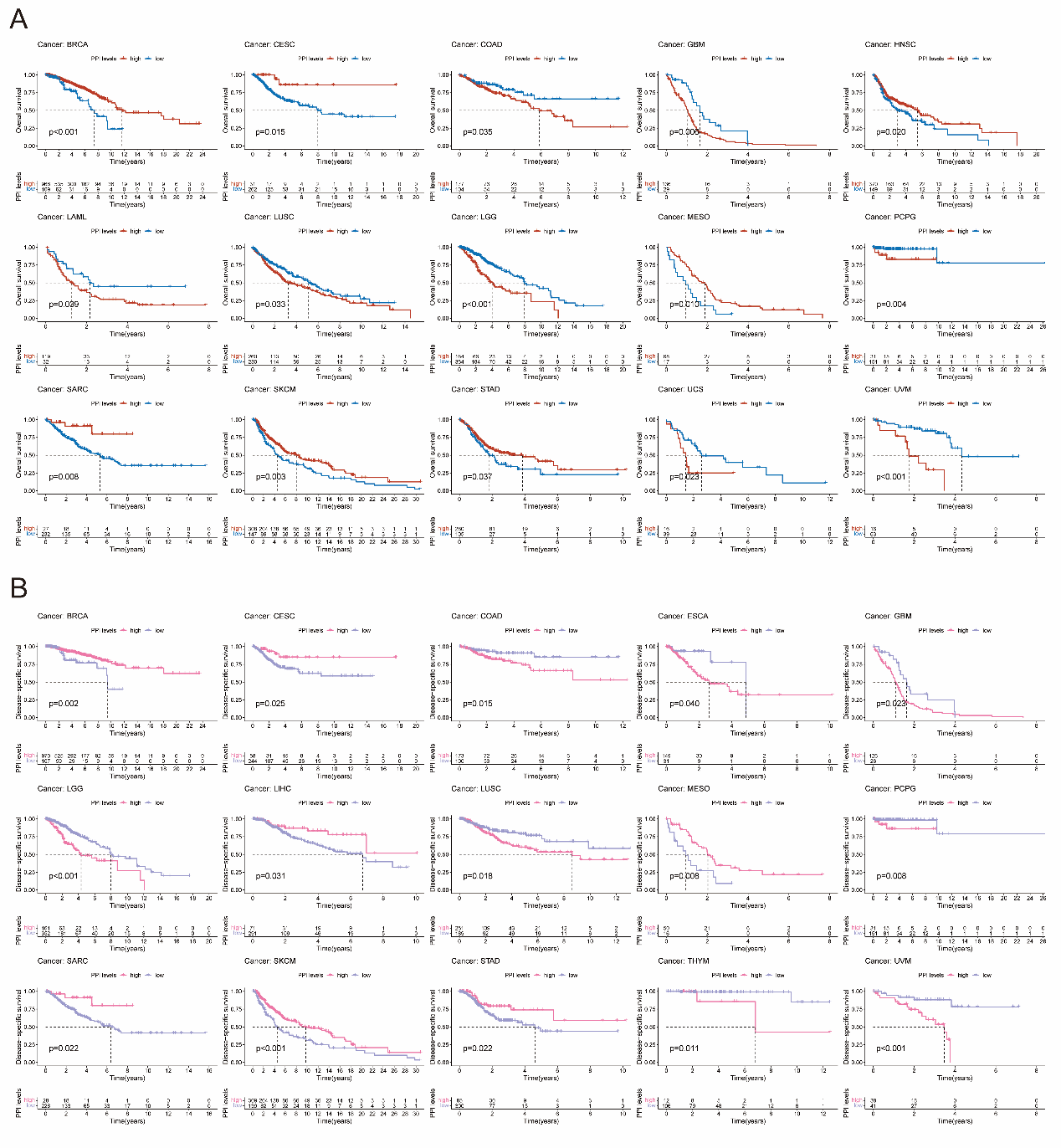


sFigure 2

A) Illustrations of predictive values for overall survival of PPI modeled by Kaplan–Meier survival curve.

B) Illustrations of predictive values for disease-specific survival of PPI modeled by Kaplan–Meier survival curve.


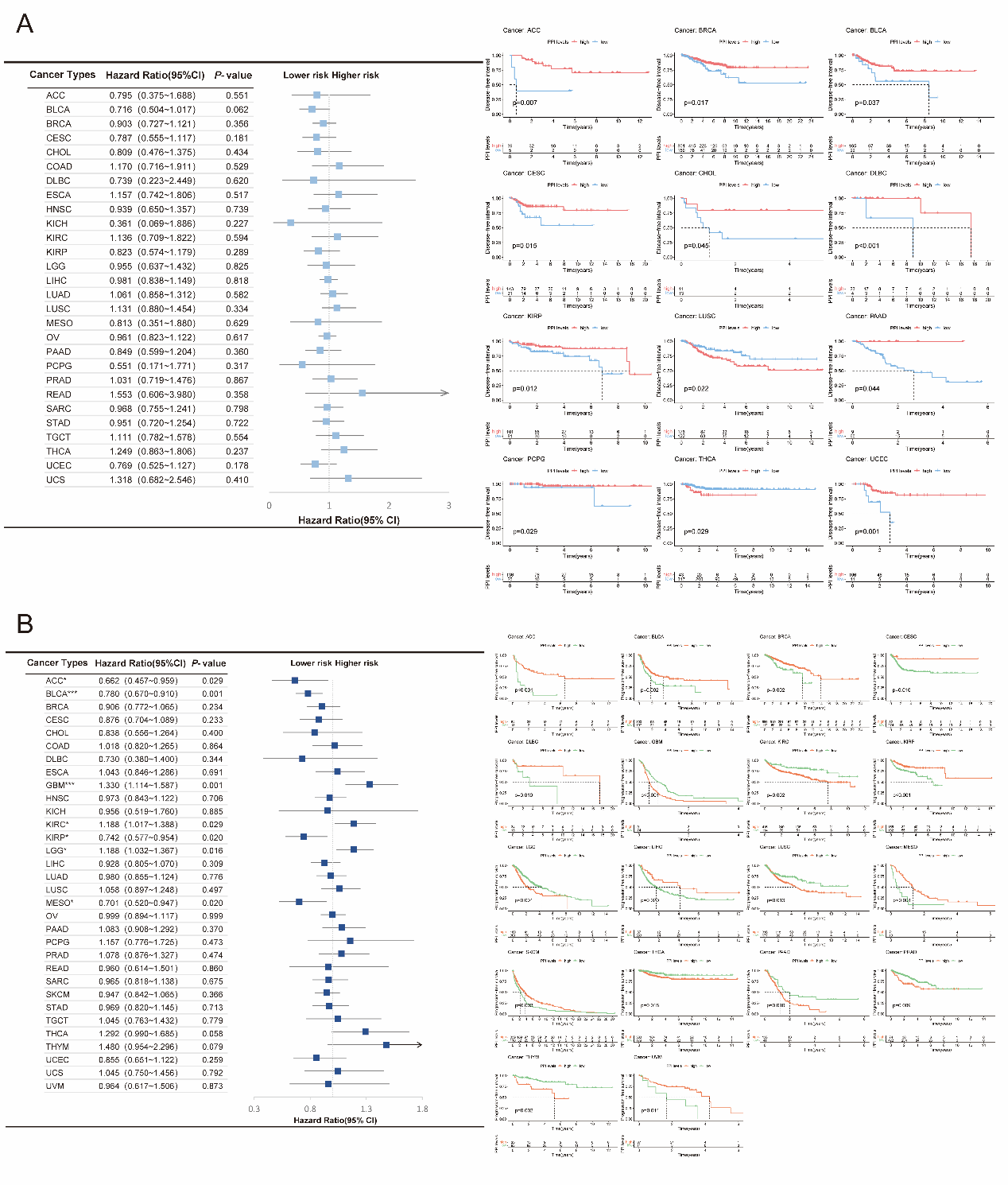


sFigure 3

A) Association between PPI and disease-free survival measured by hazard ratio (left part) and illustrations of predictive values for disease-free survival of PPI modeled by Kaplan–Meier survival curve (right part).

* p<0.05, ** p<0.01, *** p<0.001

B) Association between PPI and progression-free survival measured by hazard ratio (left part) and illustrations of predictive values for progression-free survival of PPI modeled by Kaplan–Meier survival curve (right part).

* p<0.05, ** p<0.01, *** p<0.001


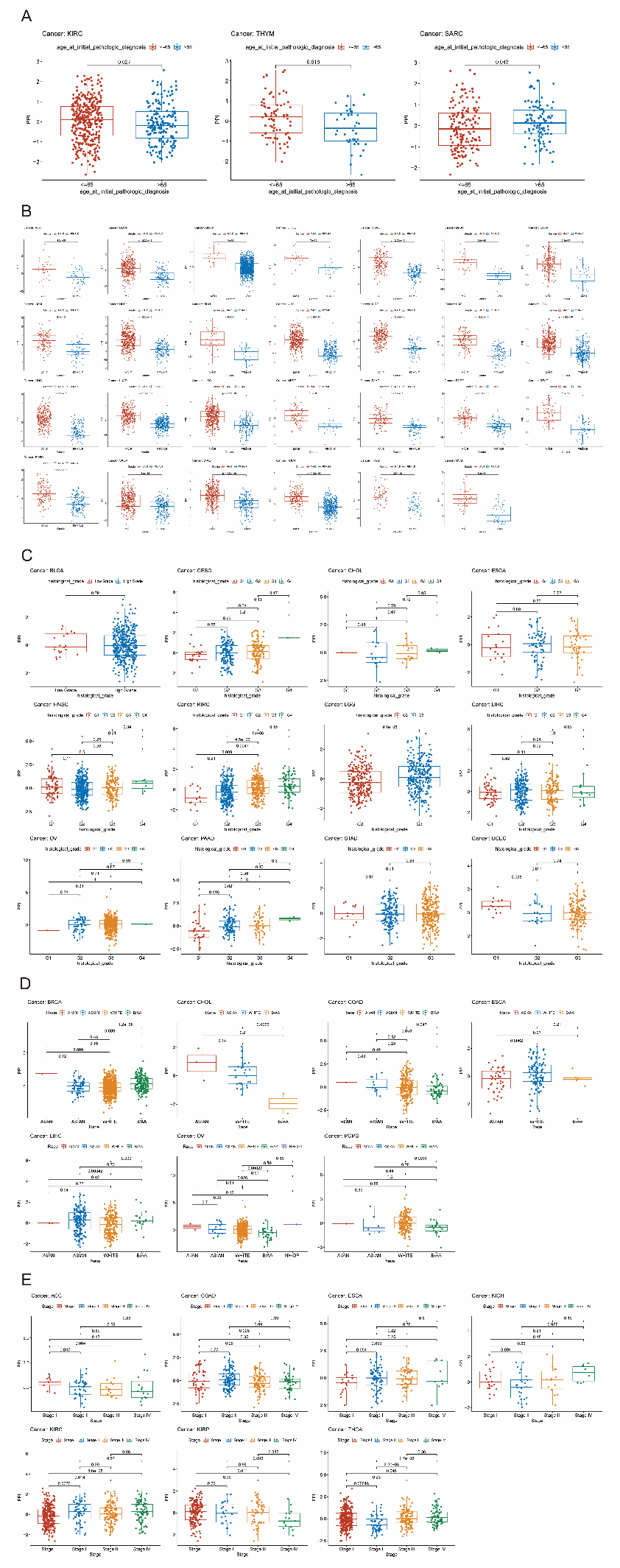


sFigure 4 Boxplots establishing the relationship between PPI and clinical characteristics in diverse cancer types, including age (A), sex (B), race (C), histologic grade (D) and stage (E).


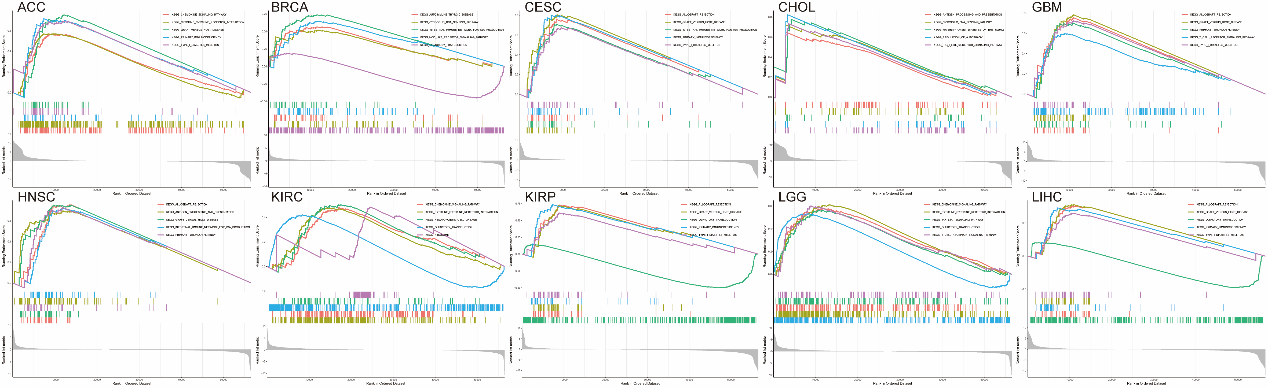


sFigure 5

KEGG analysis of PPI in ACC, BRCA, CESC, CHOL, GBM, HNSC, KIRC, KIRP, LGG and LIHC.


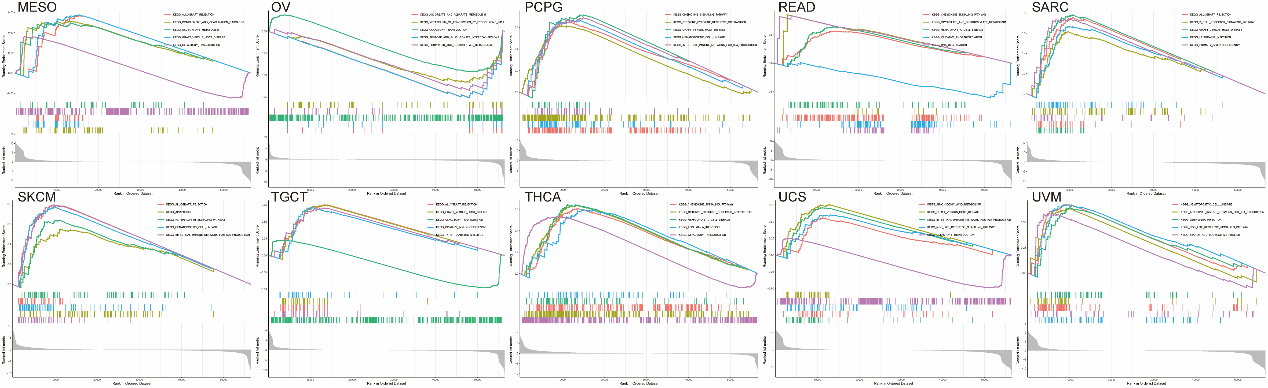


sFigure 6

KEGG analysis of PPI in MESO, OV, PCPG, READ, SARC, SKCM, TGCT, THCA, UCS, and UVM.


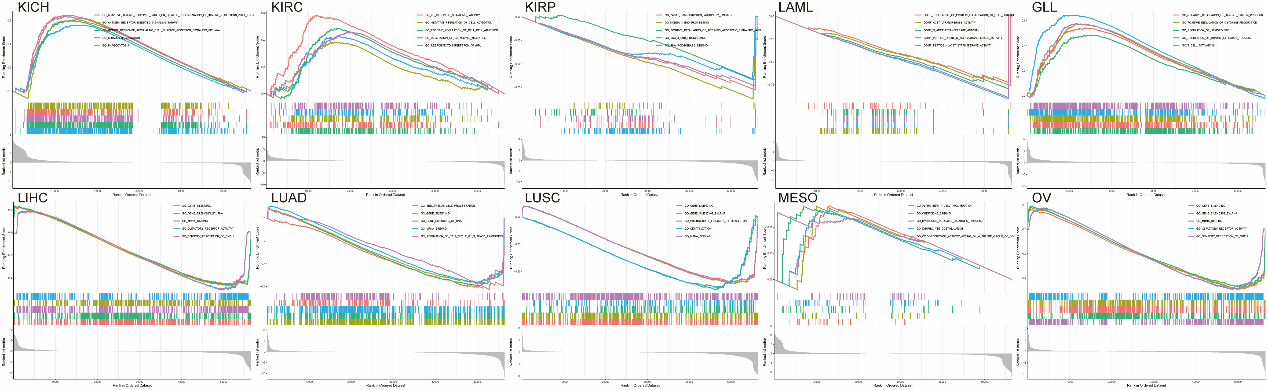


sFigure 7

GO analysis of PPI in ACC, BLCA, BRCA, CESC, CHOL, COAD, DLBC, ESCA, GBM, and HNSC.


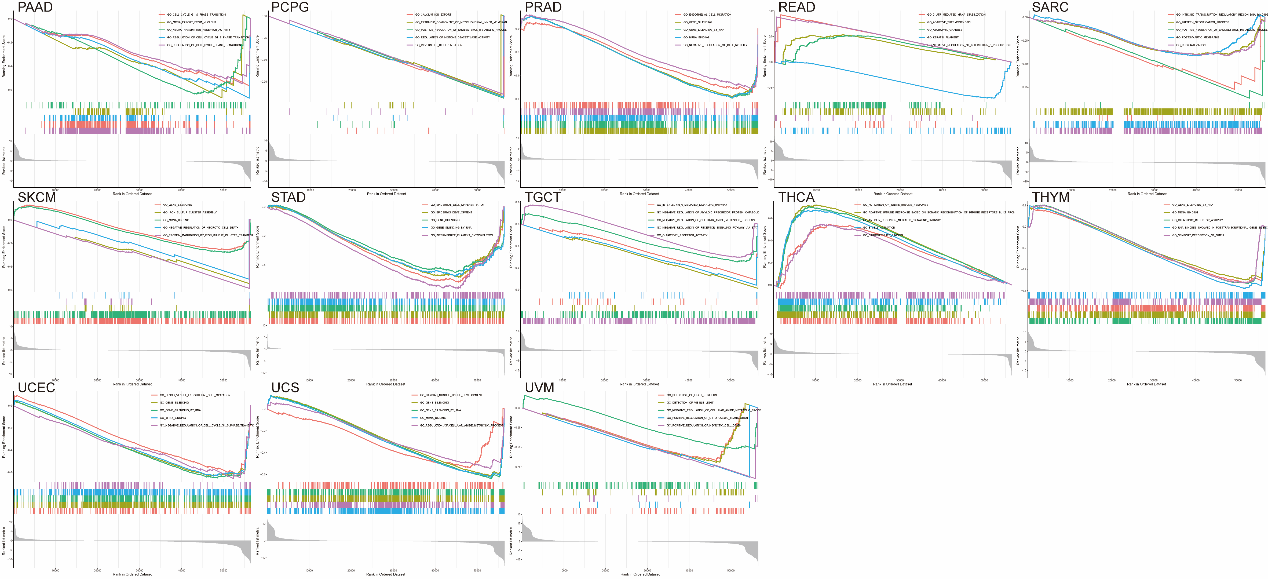


sFigure 8

GO analysis of PPI in KICH, KIRC, KIRP, LAML, GLL, LIHC, LUAD, LUSC, MESO, and OV.


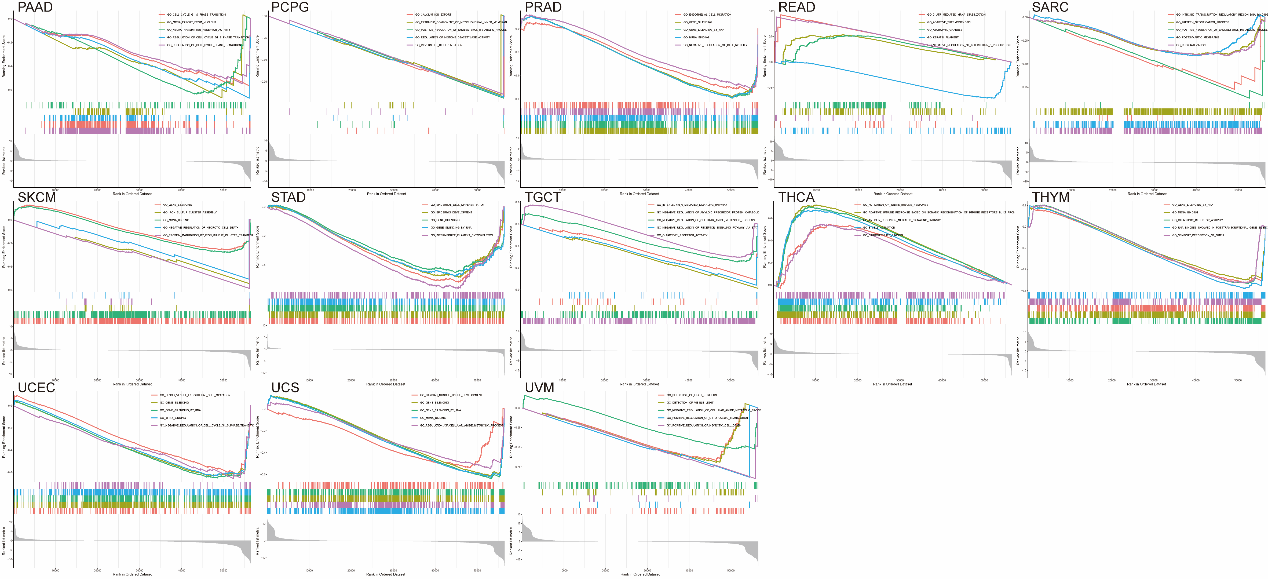


sFigure 9

GO analysis of PPI in PAAD, PCPG, PRAD, READ, SARC, SKCM, STAD, TGCT, THCA, THYM, UCEC, UCS, and UVM.


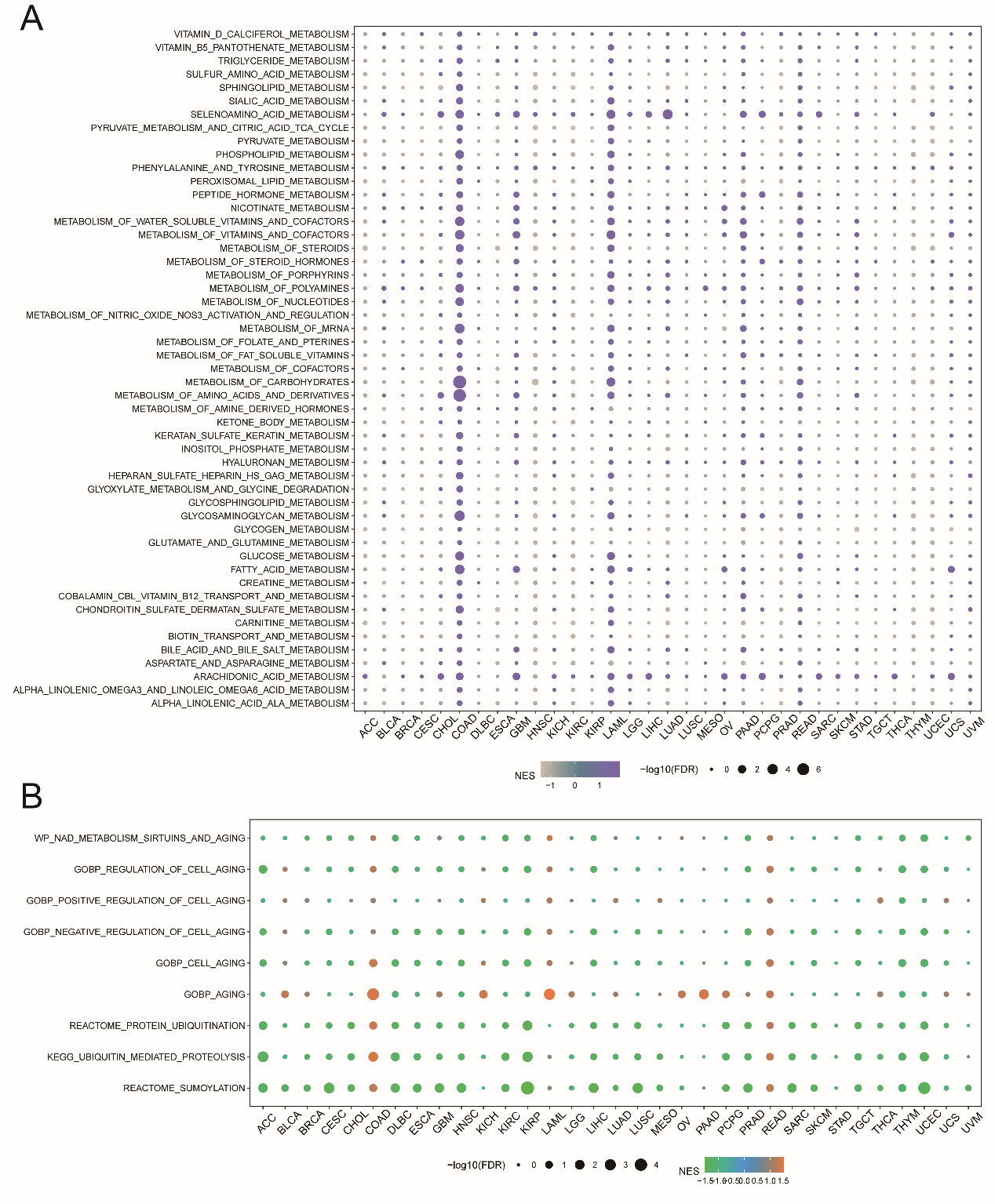


sFigure 10

A) Relation between pyroptosis and diverse metabolism pathways in various cancers.

B) Relation between pyroptosis and aging, post translational modification in various cancers.

The color presented normalized enrichment score (NES), and the size of the circle showed false discovery rate (FDR) after -log10 transformation.


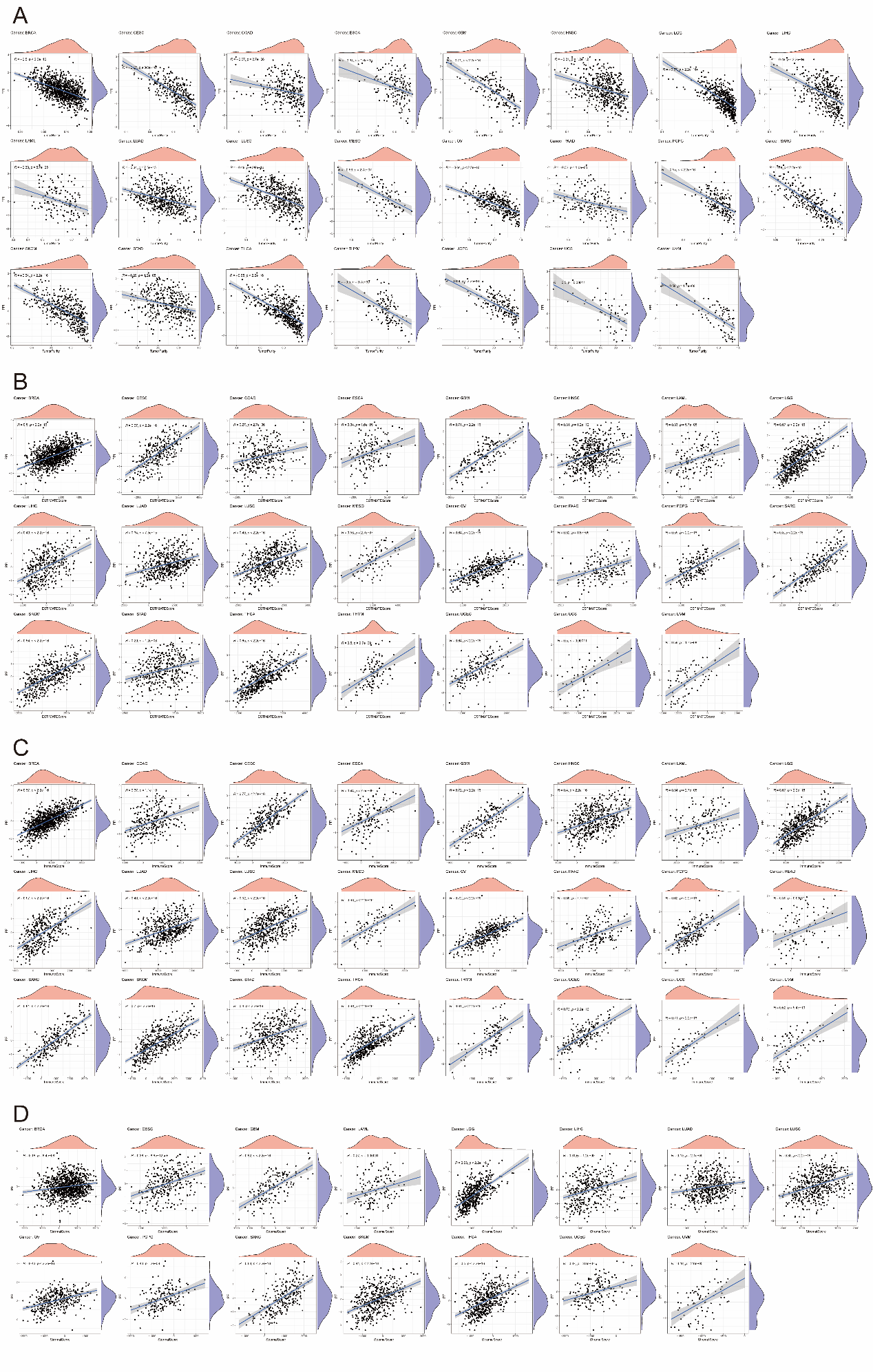


sFigure 11

Correlation of PPI to the tumor purity (A), ESTIMATE scores (B), immune scores (C), and stromal scores (D), respectively calculated with ESTIMATE package in pan-cancer.


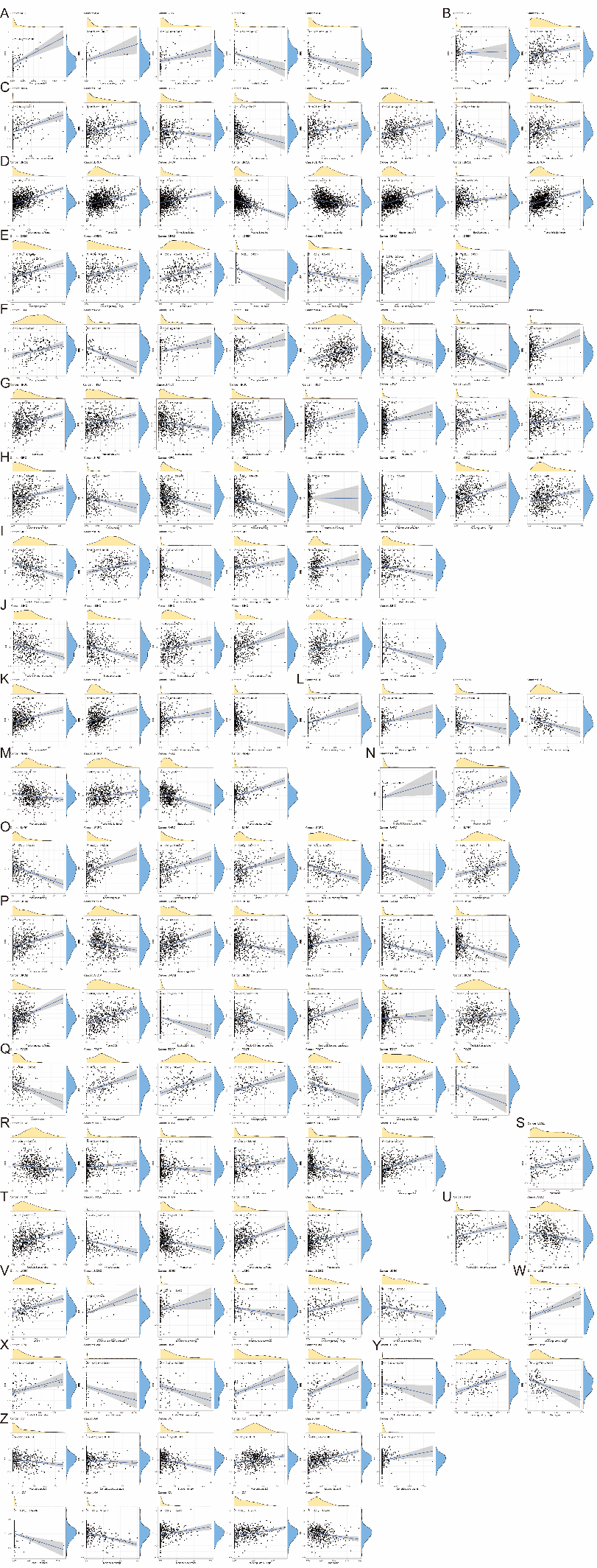


sFigure 12

Correlation between PPI and immunocyte infiltration in diverse tumor types.


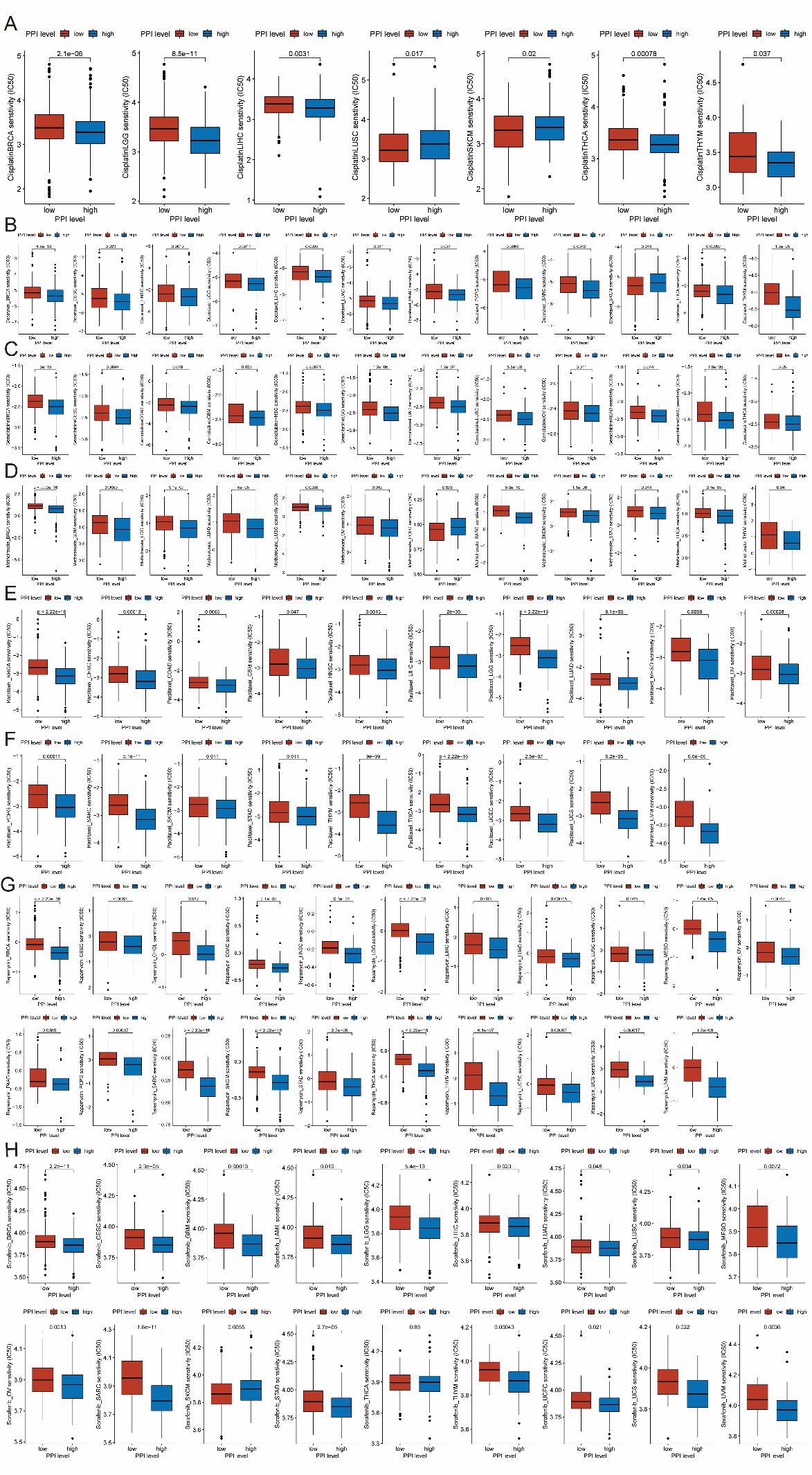


sFigure 13

Correlation between PPI and different chemotherapeutic drugs in diverse tumor types.
